# Characterization, yield optimization, scale up and biopreservative potential of fermencin SA715, a novel bacteriocin from *Lactobacillus fermentum* GA715 of goat milk origin

**DOI:** 10.1186/s12934-018-0972-1

**Published:** 2018-08-13

**Authors:** Samson Baranzan Wayah, Koshy Philip

**Affiliations:** 1 Microbiology Division, Institute of Biological Sciences, Faculty of Science, University of Malaya, Kuala Lumpur, Malaysia; 2 Department of Biochemistry, Faculty of Science, Kaduna State University, Kaduna, Nigeria

**Keywords:** Characterization, Optimization, Scale up, Biopreservation, Bacteriocin, *Lactobacillus fermentum*, Goat milk, Mode of action

## Abstract

**Background:**

Emergence of antibiotic resistance and growing consumer trend towards foods containing biopreservatives stimulated the search for alternative antimicrobials. This research is aimed at characterizing, investigating the mechanism of action, scale up optimization and evaluating the biopreservative potential of a bacteriocin from *Lactobacillus fermentum*.

**Results:**

Fermencin SA715 is a novel, broad-spectrum, non-pore-forming and cell wall-associated bacteriocin isolated from *L. fermentum* GA715 of goat milk origin. A combination of hydrophobic interaction chromatography, solid-phase extraction and reversed-phase HPLC was necessary for purification of the bacteriocin to homogeneity. It has a molecular weight of 1792.537 Da as revealed by MALDI-TOF mass spectrometry. Fermencin SA715 is potent at micromolar concentration, possesses high thermal and pH stability and inactivated by proteolytic enzymes thereby revealing its proteinaceous nature. Biomass accumulation and production of fermencin SA715 was optimum in a newly synthesized growth medium. Fermencin SA715 did not occur in the absence of manganese(II) sulphate. Tween 80, ascorbic acid, sodium citrate and magnesium sulphate enhanced the production of fermencin SA715. Sucrose is the preferred carbon source for growth and bacteriocin production. Sodium chloride concentration higher than 1% suppressed growth and production of fermencin SA715. Optimum bacteriocin production occurred at 37 °C and pH 6–7. Scale up of fermencin SA715 production involved batch fermentation in a bioreactor at a constant pH of 6.5 which resulted in enhanced production. Fermencin SA715 doubled the shelf life and improved the microbiological safety of fresh banana. Bacteriocin application followed by refrigeration tripled the shell life of banana.

**Conclusions:**

This study reveals the huge potential of fermencin SA715 as a future biopreservative for bananas and reveals other interesting characteristics which can be exploited in the preservation of other foods. Furthermore insights on the factors influencing the production of fermencin SA715 have been revealed and optimized condition for its production has been established facilitating future commercial production.

## Background

Emergence of antibiotic resistance and growing consumer trend towards foods containing biopreservatives triggered the search for alternative antimicrobials and food preservatives [1–5]. Bacteriocins produced by lactic acid bacteria (LAB) are considered potential replacements or synergists to currently used antibiotics and chemical food preservatives [4, 6, 7]. LAB bacteriocins do not significantly change the microflora of the human gastrointestinal tract [8, 9]. Moreover, they are inactivated by gut proteases, active within narrow or wide range of pH, heat-stable and their biosynthetic gene cluster is often plasmid-borne, enabling the use of genetic engineering approaches in improving production [10–14]. Studies have shown that heterologous expression of bacteriocin genes in robust hosts such as *Escherichia coli* and yeast is one of the approaches that can be employed in enhancing bacteriocin production [15, 16]. This has proved successful for pediocin PA-1 [15], bactofencin A [15] and enterocin A [16]. LAB bacteriocins are highly potent, quick-acting, possess novel modes of action and are generally recognized as safe (GRAS) facilitating both in situ and ex situ food applications [17–20].

Biopreservation is the use of microorganisms or their products and other natural bio-products to enhance safety and extend the shelf life of food mediated either by killing or reduction of the load of food spoilage microorganisms [21, 22]. This field of research is rapidly growing due to consumer inclination towards food containing biopreservatives or less synthetic chemical preservatives, fear of side effects of currently used chemical preservatives and demand for fresh-tasting and less processed food [1, 13, 23]. Fruits and vegetables are one the major sources of minerals, vitamins and fibre and are consumed worldwide [24]. Fresh fruits such as bananas have short shelf life due to their high moisture content [25]. Preservation of fresh banana is a huge task [26]. Moreover, eating them fresh exposes consumers to food-borne pathogens [27, 28]. Despite the long history of LAB bacteriocins only nisin and pediocin PA-1/AcH have gained approval for preservation of selected foods [1, 29]. Moreover, the potential of bacteriocins in the biopreservation of fresh bananas has not been investigated.

Nisin reduced the growth of *Alicyclobacillus acidoterrestris* spores in orange, grape and apple juices [1]. Growth inhibition of *Listeria monocytogenes* and *Staphylococcus aureus* in fruit juices was achieved by the application of nisin in a pulsed electric field [30]. Combined application of nisin and dimethyl dicarbonate inhibited the growth of spoilage bacteria in *Litchi* juice [31]. The anti-listerial activity of combined application of enterocin 416K1 and chitosan has been demonstrated in apples and grapes [32]. Coating of minimally processed papaya with alginate which has been incorporated with pediocin extended its shelf life [33].

Bacteriocinogenic strains of *Lactobacillus fermentum* rarely occur. These have been isolated from saliva, human vagina and green olives [34–37]. To date, bacteriocins that have been isolated from *L. fermentum* are bacteriocin L23 (estimated molecular weight ~ 7000 Da) [36], bacteriocin-like substance CS57 (estimated molecular weight > 30 kDa) [38], bacteriocin NM 332 (estimated molecular weight ~ 8000 Da) [37], BLIS LF5174 (estimated molecular weight ~ 54 kDa) [39], fermencin SD11 (33.593 kDa) [34], fermentcin B (estimated molecular weight 3–5 kDa) [40]. Of all the molecular weights of bacteriocins reportedly produced by *L. fermentum*, only one (fermencin SD11) has been accurately determined (using liquid chromatography–mass spectrometry) [34]. All other reported molecular weights are estimations.

The bacteriocin-producing potential of *L. fermentum* is highly unexplored. To date, only one *L. fermentum*-bacteriocin has been purified to homogeneity. Only limited literature on characterization of bacteriocins (purified or partially purified) from *L. fermentum* are available. Optimization studies on *L. fermentum*-derived bacteriocins are quite scarce and scale up has not been reported. Furthermore, mode of action of *L. fermentum*-derived bacteriocins has not been reported. Finally, biopreservation of fresh bananas using bacteriocin has not been investigated.

In this study a novel bacteriocin (fermencin SA715) produced by *L. fermentum* GA715 was purified to homogeneity and characterized. Its mode of action was investigated. Optimization of bacteriocin production was carried out. Scale up and enhanced production of bacteriocin was achieved by batch fermentation technique in a bioreactor at a constant pH of 6.5. An approach for biopreservation and improvement of the microbiological quality of fresh banana was demonstrated.

## Methods

### Bacterial strains and growth media

All Streptococci (except *Streptococcus mutans* GEJ11), *Bacillus cereus* ATCC 14579, *Micrococcus luteus* ATCC 10240, *Enterococcus faecium* ATCC 349 were obtained from American Type Culture Collection (ATCC). *Listeria monocytogenes* NCTC 10890 was obtained from National Collection of Type Culture (NCTC). *Streptococcus mutans* GEJ11, *Staphylococcus aureus* RF122, *Pseudomonas aeruginosa* PA7, *Corynebacterium* spp. GH17, *Escherichia coli* UT181, *Lactobacillus agilis* Yanat1, *Weissella cibaria* BavoCS3*, Enterococcus faecium* Tala1 and *Enterococcus hirae* SJ1 were taken from the culture collection of Microbial Biotechnology Laboratory, Division of Microbiology, Institute of Biological Science, Faculty of Science, University of Malaya, Kuala Lumpur, Malaysia. *Lactobacillus agilis* Yanat1, *Weissella cibaria* BavoCS3, *Enterococcus faecium* Tala1 and *Enterococcus hirae* SJ1 were maintained on MRS agar (Merck, Darmstadt, Germany). All streptococci were maintained on Todd–Hewitt agar (Difco, Le Pont de Claix, France). *Micrococcus luteus* ATCC 10240, *Bacillus cereus* ATCC 14579, *Staphylococcus aureus* RF122, *Pseudomonas aeruginosa* PA7, *Corynebacterium* spp. GH17 and *Escherichia coli* UT181 were maintained on Mueller–Hinton agar (Merck, Darmstadt, Germany). *Enterococcus faecium* ATCC 349 and *Listeria monocytogenes* NCTC 10890 were maintained on Brain–Heart infusion agar (Merck, Darmstadt, Germany). All bacterial strains were incubated at 37 °C.

### Isolation, characterization, screening of lactic acid bacteria for bacteriocin production and identification

Goat milk sample from a local manufacturer was collected in a sterile flask and fermented overnight at room temperature. De Man, Rogosa and Sharpe (MRS) broth (Merck, Darmstadt, Germany) was inoculated with the fermented goat milk and incubated overnight at 37 °C. LAB strains were isolated using MRS agar and screened for antibacterial activity using well diffusion assay as described by Wayah and Philip [41]. MRS broth cultures incubated overnight were centrifuged (10,000×*g*) at room temperature and supernatant was collected and passed through 0.22 µm Millipore filter to obtain cell-free supernatant (CFS). Antibacterial activity of CFS (40 µl) against main targets of interest (*Micrococcus luteus* ATCC 10240, *Corynebacterium* spp. GH17, *Bacillus cereus* ATCC 14579, *Pseudomonas aeruginosa* PA7 and *Escherichia coli* UT181) was tested. Microscopic observation, catalase and oxidase tests were performed. Molecular identification of bacteriocinogenic LAB strain was carried out by amplifying and sequencing the 16S rRNA gene using the universal primer 27 and 1492R. A homology search of the 16S rRNA gene was carried out using the NCBI data base (https://blast.ncbi.nlm.nih.gov/Blast.cgi).

### Purification of fermencin SA715 and determination of molecular weight

In order to purify the bacteriocin to homogeneity the bacteriocin producer was grown in MRS broth for 18 h at 37 °C and centrifuged (10,000×*g* for 20 min at 4 °C) to collect the supernatant which was subsequently filtered using 0.22 µm filter to obtain CFS. This was subjected to hydrophobic interaction chromatography (HIC) in which methanol (Merck, Darmstadt, Germany) gradient (50–95%) was used for elution of the peptides adsorbed onto the surfaces of amberlite XAD-16 particles (Sigma-Aldrich, St. Louis, USA) packed in a glass column. Fractions were evaporated and antibacterial activity was determined using well diffusion assay. The active fraction was further purified using solid-phase extraction method in which Strata C18-E column was used. Acetonitrile (ACN) gradients (10%, 20%, 30% and 50%) were used for elution of bacteriocin. The active fraction was subjected to reversed-phase HPLC (RP-HPLC, Waters, USA). The mobile phase consisted of two solvents: A (95% Mili-Q water (Millipore, USA) and 5% ACN (Merck, Germany) and B (100% ACN). Elution was done using the biphasic ACN gradient at a flow rate of 1 ml/min over 60 min. Fractions were collected and evaporated using a centrifugal vacuum evaporator (Thermofisher Scientific, Vantaa, Finland). Antibacterial activity was tested. Molecular weight of the bacteriocin was determined by subjecting the active HPLC fraction to matrix-assisted laser desorption-ionization time-of-flight (MALDI-TOF) mass spectrometry.

### Antibacterial spectrum and MIC

This experiment was done to determine the antibacterial spectrum of fermencin SA715. Active fraction from HIC was used for this investigation which was achieved via well diffusion assay as described by Wayah and Philip [41]. Minimum inhibitory concentration (MIC) of main bacterial targets of interest (*Micrococcus luteus* ATCC 10240, *Corynebacterium* spp. GH17, *Bacillus cereus* ATCC 14579 *Pseudomonas aeruginosa* PA7 and *Escherichia coli* UT181) was determined by using the broth microdilution assay as described by Mota-Meira et al. [42] with little modifications. Twofold dilutions of pure fermencin SA715 were prepared in adequate media and 50 µl of each was added to 96-well microtiter plate. Overnight culture of indicator bacteria was diluted to OD_600nm_ = 0.1 and 50 µl was added to each pure bacteriocin preparation. Wells containing indicator bacterial strain without bacteriocin were used as positive control while wells containing only the media were used as blank. Incubation was done at 37 °C and optical density at 600 nm was monitored with a microplate reader (Thermo Scientific Model: Multiskan GO, Finland) over a period of 24 h. MIC was defined as the concentration of the bacteriocin which caused growth reduction by more than 80% compared with the positive control.

### Stability test for fermencin SA715

Stability of fermencin SA715 to heat, pH and treatments with proteolytic enzymes was investigated as described in our previous publication [41]. Heat stability was performed by subjecting fermencin SA715 to temperatures of 40, 50, 60, 70, 80, 90 and 100 °C for 30 min and subsequently testing its residual antibacterial activity. Different pH values ranging from 2 to 10 were used to test the effect of pH on the antibacterial activity of the bacteriocin. The mixture was incubated for 2 h at 25 °C and residual antibacterial activity was tested. The bacteriocin was added to different enzymes (proteinase K, lysozyme, α-chymotrypsin, protease, catalase, trypsin and peptidase) so that the final concentrations of the enzymes reached 1 mg/ml. The mixtures were incubated at 37 °C for 1 h before testing for residual antibacterial activity.

### Pore formation assay

To investigate the mode of action of fermencin SA715, *Micrococcus luteus* was grown until it attained an OD_600nm_ = 0.6. Bacterial cells were obtained by centrifuging at 2000×*g* for 10 min and washed three times using sterile 10 mM sodium phosphate buffer pH 7.2. The cell pellet was re-suspended in 5 ml of the same buffer and mixed with 5 μl of SYTOX^®^ Green dye. Ninety microliters (90 μl) of the stained bacteria was mixed with 10 μl of fermencin SA715 (5× MIC). Positive control used in this study was nisin while the negative control was stained bacterial cells without fermencin SA715 addition. The plate was sealed by using adhesive cover and then placed in the Step One Plus real-time PCR system (Applied Biosystems, USA) and fluorescence was monitored for 30 min.

### Bacteriocin-cell wall association assay

This experiment was done to understand the extent of association of the bacteriocin with the cell wall of its producer. Ten percent 24-h MRS broth culture was added to fresh MRS broth and incubated at 37 °C for 18 h after which it was centrifuged at 10,000×*g* for 20 min at 4 °C. Antibacterial activity of CFS was tested using well diffusion assay. The cell pellet was re-suspended in 95% (v/v) methanol (Merck, Darmstadt, Germany), pH 2 and stirred overnight at 4 °C on a magnetic stirrer (Benchmark, Edison NJ, USA). The cell suspension was centrifuged (10,000×*g* for 30 min at 4 °C) and the supernatant was filtered using a filter (Millipore 0.22 µm) after which methanol was evaporated on a water bath (Memmert, Schwalbach, Germany) at 40 °C. The cell extract was reconstituted in ultrapure water and antibacterial activity was tested.

### Optimization of fermencin SA715 production

#### Preparation of inoculum

A 24-h old MRS broth culture of *L. fermentum* GA715 was centrifuged (2000×*g* for 10 min) to collect the cell pellet which was re-suspended in sterile phosphate buffered saline (PBS) and washed by centrifuging twice at the same condition each time adding fresh sterile PBS. After the second wash, cell pellet was re-suspended in PBS and adjusted to an optical density (OD) of 0.1 at 600 nm.

#### Influence of media on production of fermencin SA715

Bacterial suspension was added (10%) to four different media three of which are newly synthesized media and the other is MRS broth. The new media compositions included SGSL: 1% tryptone (Difco, Le Ponte de Claix, France), 1% peptone, (Difco, Le Ponte de Claix, France), 1% yeast extract (Difco, Le Ponte de Claix, France), 5% glucose (Merck, Darmstadt, Germany), 0.05% ascorbic acid (R & M Chemicals, Essex, UK), 0.2% sodium citrate (Peking Chemical Works, Peking, China), 0.005% manganese(II) sulphate (BDH Chemicals Ltd, Poole, England), 0.025% magnesium sulphate (Halewood Chemical Ltd, Middlesex, England), 0.02% sodium chloride (John Kollin Corporation, USA) and 0.1% Tween 80 (Sigma-Aldrich, Missouri, USA), TPYGMA: 1% tryptone, 1% peptone, 1% yeast extract, 5% glucose, 1% 2-morpholinoethanesulphonic acid, MESA (Merck, Darmstadt, Germany) and 0.05% ascorbic acid, TPYGCaT80: 1% tryptone, 1% peptone, 1% yeast extract, 5% glucose, 0.1% CaCO_3_ and 0.1% Tween 80. Incubation at 37 °C was done after broth inoculation. OD was monitored over a period of 24 h. Fermencin SA715 production was determined by well diffusion assay using *Micrococcus luteus* as the bacterial target.

#### Effect of various components of SGSL on bacterial growth and production of fermencin SA715

Inoculum was added (10%) to SGSL and nine of its derivatives. These are: S1 (SGSL), S2 (SGSL without Tween 80), S3 (SGSL without tryptone), S4 (SGSL without peptone), S5 (SGSL without yeast extract), S6 (SGSL without NaCl), S7 (SGSL without ascorbic acid), S8 (SGSL without sodium citrate), S9 (SGSL without MnSO_4_), S10 (SGSL without MgSO_4_). OD and zone of inhibition (ZOI) was measured after incubation at 37 °C for 24 h. *Micrococcus luteus* was used as the target bacterial strain.

#### Effect of carbon source on bacterial growth and production of fermencin SA715

Ten percent inoculum was added to SGSL (S1 containing 5% glucose as carbon source) and its derivatives containing various carbon sources. These are S11 (SGSL containing 5% sucrose as carbon source), S12 (SGSL containing 5% galactose as carbon source), S13 (SGSL containing 5% fructose as carbon source), S14 (SGSL containing 5% lactose as carbon source) and S15 (SGSL containing 5% maltose as carbon source) followed by incubation at 37 °C for 24 h. Growth and bacteriocin titer was determined afterwards.

#### Effect of sodium chloride concentration on bacterial growth and production of fermencin SA715

Various derivatives of SGSL containing different concentrations of sodium chloride (0.02%, 0.5%, 1%, 2% and 4%) were inoculated with 10% inoculum and incubated at 37 °C for 24 h followed by measurements of OD and ZOI.

#### Effect of pH and incubation temperature on bacterial growth and production of fermencin SA715

MRS broth was adjusted to various pH values (3, 4, 5, 6, 7, 8 and 9) before autoclaving. It was inoculated (10%) with the prepared bacterial suspension and incubated at 37 °C for 24 h followed by measurements of OD and zone of inhibition.

The same concentration of inoculum was added to MRS broth and incubated at various temperatures (25, 32, 37, 40 and 46 °C). Bacterial growth and production of fermencin SA715 was measured after 24 h of incubation.

### Scale up of fermencin SA715 production using batch fermentation

An 18-h old MRS broth culture of *L. fermentum* GA715 was centrifuged (2000×*g* for 10 min) to collect the cell pellet which was re-suspended in sterile S11 (SGSL containing 5% sucrose as carbon source) and washed by centrifuging twice at the same condition each time adding fresh sterile S11. After the second wash, cell pellet was re-suspended in S11 and adjusted to an optical density (OD) of 0.1 at 600 nm. Ten percent of this inoculum was added to sterile S11 in a bioreactor (Sartorius Stedim, Germany). Bioreactor parameters were set as follows: temperature of 37 °C, stirrer speed of 200 rpm and pH value of 6.5. Antifoam was not added because there was no significant foaming during fermentation. Growth and bacteriocin production was monitored.

### Biopreservation of banana

In order to investigate the biopreservative potential of fermencin SA715 bacteriocin preparation (2.0714 mM) was topically applied to mature, fresh banana samples. This was achieved by soaking sterile cotton swab in the bacteriocin preparation and gently rubbing the surfaces of banana samples with it. Some fermencin SA715-treated banana samples were kept at ambient condition while others were refrigerated (4 °C). Non-fermencin SA715-treated banana samples kept at ambient condition and at refrigeration condition (4 °C) served as control samples. Samples were monitored for morphological changes. At the onset of deterioration of control samples, sterile cotton swabs were used to collect surface microflora of both control and bacteriocin-treated banana samples and bacterial count (CFU/ml) was determined. The experiment was allowed to proceed until the onset of deterioration of bacteriocin-treated banana samples. Shelf-life was measured. Experiments were done in triplicates.

### Data analysis

Data was subjected to one-way analysis of variance using SPSS software (version 22). Results were expressed as means of three replications. Mean separation was done using least significant difference.

## Results

### Isolation, characterization, screening of lactic acid bacteria (LAB) for bacteriocin production and identification

LAB strains were successfully isolated from goat milk. They are Gram positive, catalase and oxidase negative. The LAB strain with the strongest antibacterial activity against the main targets of interest (*Micrococcus luteus* ATCC 10240, *Corynebacterium* spp. GH17, *Bacillus cereus* ATCC, *Pseudomonas aeruginosa* PA7 and *Escherichia coli* UT181) was selected and identified based on 99% similarity with a strain of *L. fermentum* in NCBI database. We subsequently named our strain *L. fermentum* GA715.

### Purification of fermencin SA715 and determination of molecular weight

**Fig. 1:**
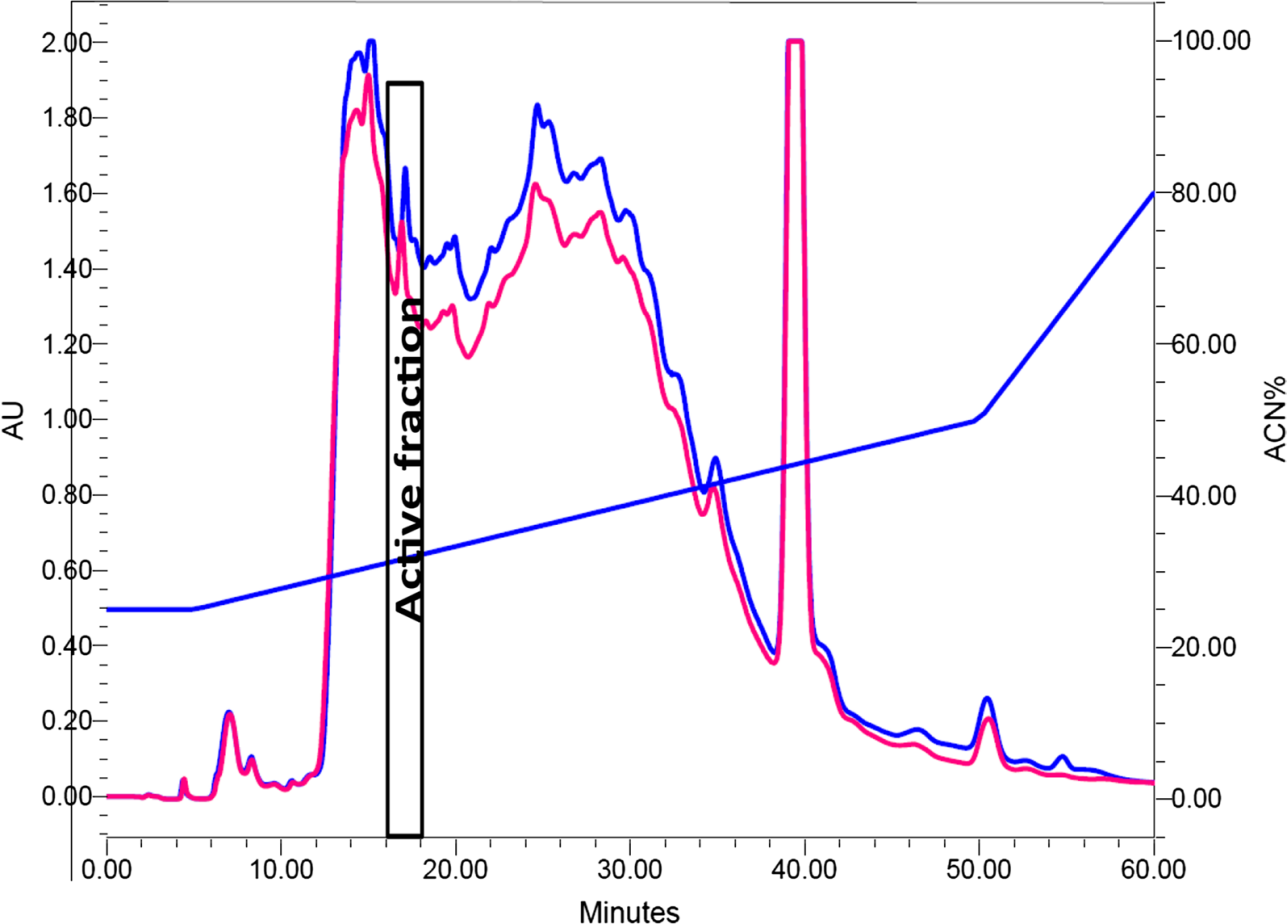
Reversed-phase HPLC chromatogram of active fraction from solid-phase bacteriocin extraction. The vertical lines indicate the retention time. Active fraction from solid-phase extraction was subjected to RP-HPLC

**Fig. 2:**
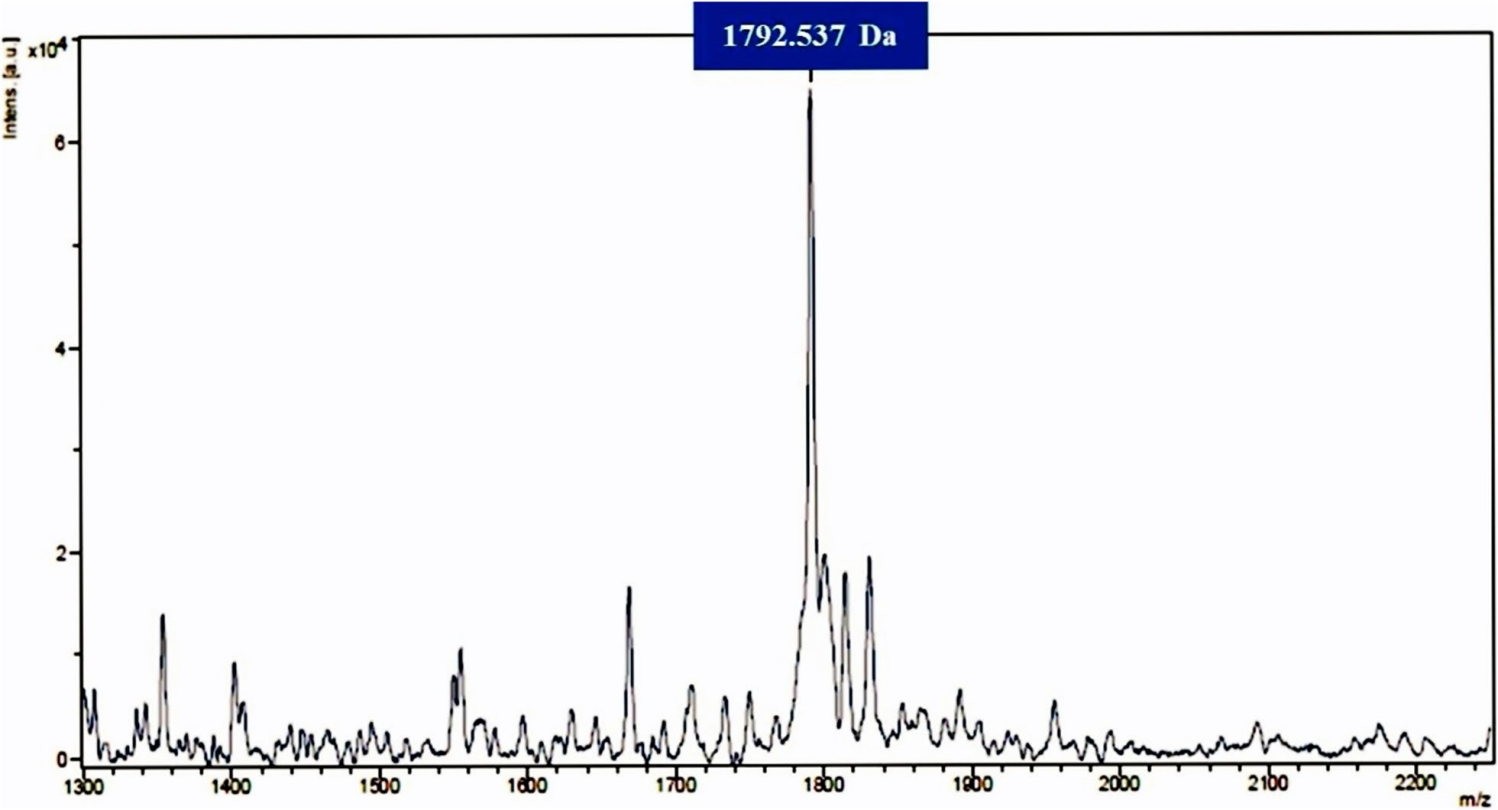
MALDI-TOF mass spectrum of purified fermencin SA715

Bacteriocin was purified to homogeneity by the sequential use of HIC, solid-phase extraction and RP-HPLC. It was eluted at a retention time of 17–19 min (Fig. 1). MALDI-TOF mass spectrometry of purified bacteriocin showed that the molecular weight is 1792.537 Da (Fig. 2).

### Antibacterial spectrum and MIC

Fermencin SA715 was strongly inhibitory towards selected bacterial targets belonging to various genera showing its broad antibacterial spectrum. These include *Micrococcus luteus* ATCC 10240, *Corynebacterium* spp. GH17, *Pseudomonas aeruginosa* PA7, *Staphylococcus aureus* RF122, *Listeria monocytogenes* NCTC 10890, *Streptococcus mutans* GEJ11, *Streptococcus equisimilis* ATCC 12388, *Streptococcus sanguinis* ATCC 10556, *Bacillus cereus* ATCC 14579 and *Escherichia coli* UT181 (Table 1). It was not inhibitory against *Streptococcus pyogenes* ATCC 12344 and *Weissella cibaria* BavoCS3. *Micrococcus luteus* ATCC 10240 had the lowest MIC value (8.93 µM) while the highest was against *Staphylococcus aureus* RF122 (103.57 µM) and *Escherichia coli* UT181 (103.57 µM) (Table 2).

**Table 1:** Antibacterial spectrum of fermencin SA715

Indicator	Zone of inhibition (mm)
*Streptococcus mutans* GEJ11	+++
*Streptococcus pyogenes* ATCC 12344	−
*Streptococcus equisimilis* ATCC 12388	+++
*Streptococcus sanguinis* ATCC 10556	+++
*Listeria monocytogenes* NCTC 10890	++++
*Staphylococcus aureus* RF122	++++
*Bacillus cereus* ATCC 14579	+++
*Corynebacterium* spp. GH17	++++
*Pseudomonas aeruginosa* PA7	++++
*Escherichia coli* UT181	+++
*Micrococcus luteus* ATCC 10240	++++
*Lactobacillus agilis* Yanat1	+++
*Enterococcus faecium* ATCC 349	++
*Enterococcus faecium* Tala1	+++
*Weissella cibaria* BavoCS3	−
*Enterococcus hirae* SJ1	++++

The antibacterial spectrum of fermencin SA715 was determined using active fraction from HIC

++++, inhibition zone > 20 mm; +++, inhibition zone 15–20 mm; ++, inhibition zone < 15 mm; −, no inhibition

**Table 2:** Minimum inhibitory concentration for fermencin SA715 against selected bacterial targets

Indicator	MIC (µM)
*Pseudomonas aeruginosa* PA7	51.79
*Micrococcus luteus* ATCC 10240	8.93
*Escherichia coli* UT181	103.57
*Staphylococcus aureus* RF122	103.57
*Corynebacterium* spp. GH17	12.95

Purified bacteriocin was used in the determination of MIC values of targets of interest

### Stability test for fermencin SA715

The zones of inhibition obtained after treatment of fermencin SA715 at 40–90 °C were comparable to that of the control (un-treated) (Table 3). Slightly lower ZOI was obtained after treatment at 100 °C. There was no change in ZOI upon treatment of fermencin SA715 with lysozyme, lyticase and catalase but a significant change in ZOI was observed after treatment with proteinase K, peptidase, trypsin, α-chymotrypsin and proteinase (Table 3). Treatment at pH 2–7 caused a slight decrease in ZOI while a significant drop was observed due to treatment at pH value ranging from 8 to 10 (Table 3).

**Table 3:** Stability tests for fermencin SA715 (heat, enzyme and pH)

Test	Zone of inhibition (mm)
Control	++++
Heat	
40	++++
50	++++
60	++++
70	++++
80	++++
90	++++
100	+++
Enzyme	
Proteinase K	++
Lysozyme	++++
Peptidase	++
Lyticase	++++
Catalase	++++
Trypsin	+++
α-Chymotrypsin	+++
Protease	+++
pH	
2–7	+++
8	++
9 and 10	+

++++, inhibition zone > 20 mm; +++, inhibition zone 16–20 mm; ++, inhibition zone = 15 mm; +, inhibition zone < 15 mm

### Pore formation assay

**Fig. 3:**
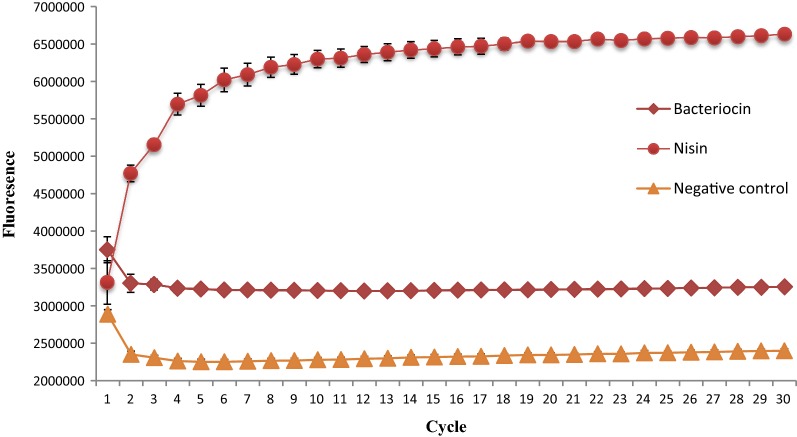
Membrane permeabilization assay using *Micrococcus luteus* ATCC 10240 as indicator. Mode of action of fermencin SA715 was investigated using pore-formation assay in which SYTOX^®^ Green dye was used in staining of leaked intracellular DNA

Treatment of the stained bacterial target with fermencin SA715 did not cause increase in fluorescence indicating that there was no increase in DNA concentration. Similar result was obtained for the un-treated bacterial cells. However, nisin caused increase in fluorescence indicating increase in DNA concentration due to leakage of intracellular DNA (Fig. 3).

### Bacteriocin-cell wall association assay

Of the total activity (100%), 30.94% was observed in the CFS while 69.06% was seen in the cell extract (Table 4). This assay gives insight on the extent of adherence of bacteriocin to the cell wall of its producer.

**Table 4:** Bacteriocin recovered from cell-free supernatant and cell extract of *L. fermentum* GA715

Source of bacteriocin	ZOI (mm)	Activity (%)
Cell-free supernatant	14.17 ± 0.67	30.94
Cell extract	31.63 ± 1.15	69.06
Total	45.80	100.00

Bacteriocin-cell wall association assay was conducted by determining antibacterial activity of cell extract and CFS

### Optimization of fermencin SA715 production

#### Influence of media on production of fermencin SA715

**Fig. 4:**
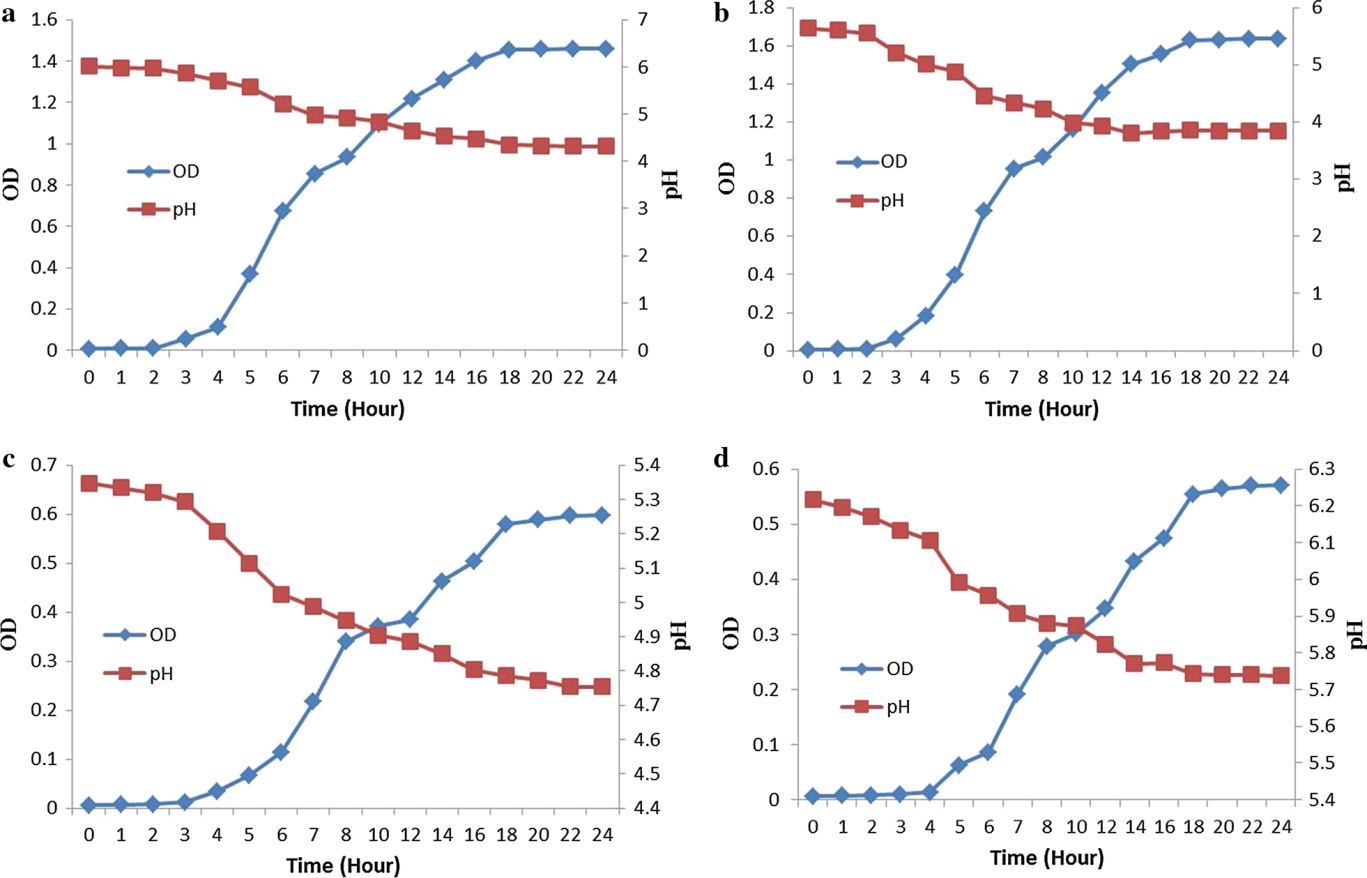
Growth of *L. fermentum* GA715 and pH changes in different media. **a** MRS; **b** SGSL; **c** TPYGMA; **d** TPYGCaT80

The OD values obtained after 24 h of growth were: 1.636 (SGSL), 1.460 (MRS), 0.597 (TPYGMA) and 0.570 (TPYGCaT80) (Fig. 4). The final pH values of broth culture were: 3.84, 4.32, 4.75 and 5.74 corresponding to SGSL, MRS, TPYGMA and TPYGCaT80 respectively (Fig. 4). Fermencin SA715 production was observed in SGSL (ZOI value of 15.23 mm) and MRS (ZOI value of 12.28 mm) but not in TPYGMA and TPYGCaT80. SGSL had significantly higher (p < 0.05) bacteriocin titer compared to MRS.

#### Effect of various components of SGSL on bacterial growth and production of fermencin SA715

**Fig. 5:**
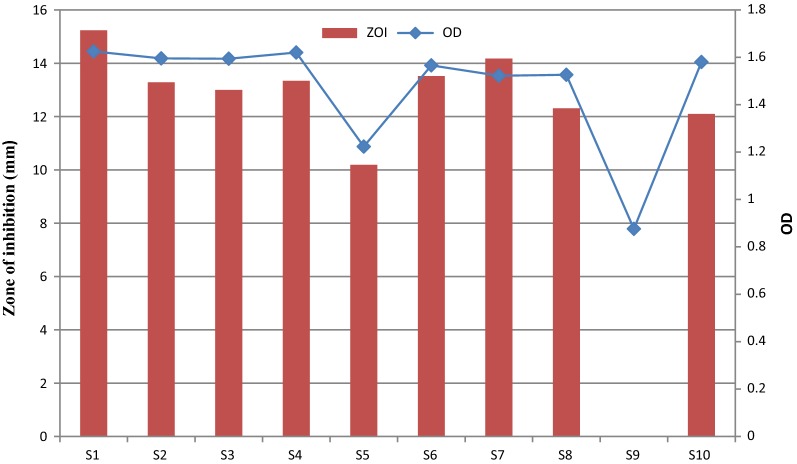
Effect of various components of SGSL on bacterial growth and production of fermencin SA715. **S1** (SGSL), **S2** (SGSL without Tween 80), **S3** (SGSL without tryptone), **S4** (SGSL without peptone), **S5** (SGSL without yeast extract), **S6** (SGSL without NaCl), **S7** (SGSL without ascorbic acid), **S8** (SGSL without sodium citrate), **S9** (SGSL without MnSO_4_), **S10** (SGSL without MgSO_4_)

SGSL (S1, control) had the highest ZOI compared to its derivatives (p < 0.05). Removal of any single component of the SGSL medium caused a significant drop in ZOI (Fig. 5). ZOI values for S2 (SGSL without Tween 80), S4 (SGSL without peptone), S6 (SGSL without NaCl) and S7 (SGSL without ascorbic acid) were not significantly different. ZOI obtained for S3 (SGSL without tryptone), S8 (SGSL without sodium citrate) and S10 (SGSL without MgSO_4_) were not significantly different but were significantly different (p < 0.05) from that obtained for S5 (SGSL without yeast extract). There was no fermencin SA715 production in S9 medium (SGSL without MnSO_4_). OD values for S1, S2, S3, S4, S6 and S10 were not significantly different (p > 0.05). OD obtained for S6 and S10 were not significantly different from S7 and S8. OD at S5 was higher than S9 which was the least OD value obtained. Modification of SGSL medium also had effect on the initial pH of the uninoculated broth and final pH of the culture.

#### Effect of carbon source on bacterial growth and production of fermencin SA715

**Fig. 6:**
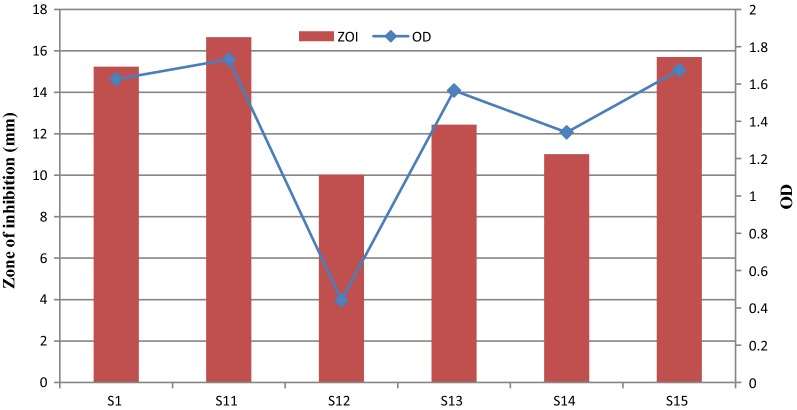
Effect of carbon source on bacterial growth and production of fermencin SA715. **S1** (SGSL containing 5% glucose as carbon source), **S11** (SGSL containing 5% sucrose as carbon source), **S12** (SGSL containing 5% galactose as carbon source), **S13** (SGSL containing 5% fructose as carbon source), **S14** (SGSL containing 5% lactose as carbon source) and **S15** (SGSL containing 5% maltose as carbon source)

The highest growth and ZOI was obtained in S11 (SGSL containing sucrose as a carbon source) while the lowest was obtained in S12 (SGSL containing galactose as a carbon source) (Fig. 6). All treatments (S1, S11, S12, S13, S14 and S15) were significantly different (p < 0.05). Initial pH of the uninoculated broth and the final pH of the culture varied for SGSL and its derivatives.

#### Effect of sodium chloride concentration on bacterial growth and production of fermencin SA715

**Fig. 7:**
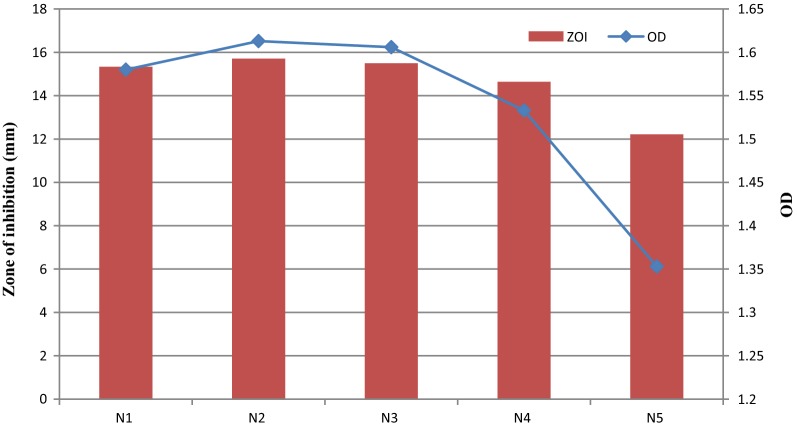
Effect of sodium chloride concentration on bacterial growth and production of fermencin SA715. **N1** (0.02% NaCl), **N2** (0.5% NaCl), **N3** (1% NaCl), **N4** (2% NaCl), **N5** (4% NaCl)

Bacteriocin production at 0.02%, 0.5% and 1% was not significantly different (p > 0.05) but significantly higher than at 2% and 4% which had the least ZOI (Fig. 7). There was a significant increase in growth (p < 0.05) as sodium chloride concentration was increased from 0.02 to 1% after which OD and ZOI declined with increase in sodium chloride concentration (Fig. 7). OD at 0.5% and 1% were not significantly different (p > 0.05). Final pH of culture varied but there were no variations in the initial pH of the uninoculated broth.

#### Effect of pH and incubation temperature on bacterial growth and production of fermencin SA715

**Fig. 8:**
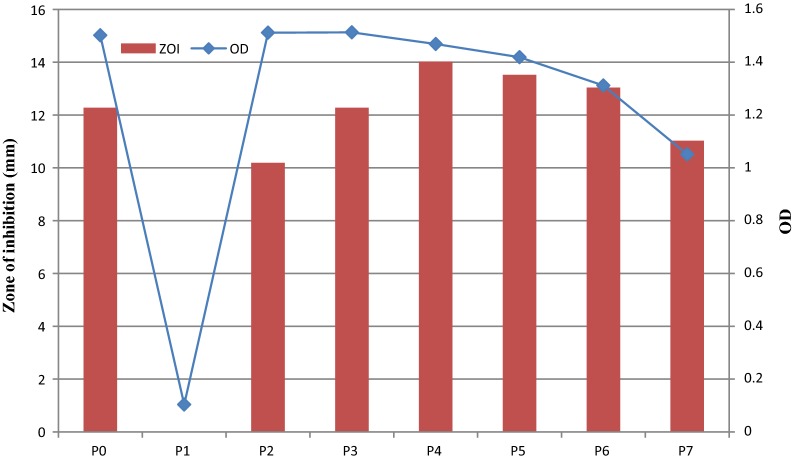
Effect of pH on bacterial growth and production of fermencin SA715. **P0** (unadjusted pH, control), **P1** (pH 3), **P2** (pH 4), **P3** (pH 5), **P4** (pH 6), **P5** (pH 7), **P6** (pH 8), **P7** (pH 9)

There was no fermencin SA715 production at pH 3 (Fig. 8). ZOI for pH 6 and 7 were not significantly different (p > 0.05). ZOI values for pH 7 and pH 8 were not significantly different but higher than that obtained for unadjusted MRS and pH 5. ZOI for pH 9 and pH 4 (the least) were significantly different. OD values for unadjusted MRS, pH 4 and pH 5 were not significantly (p > 0.05) but were significantly higher (p < 0.05) than those obtained at other pH values. pH 3 had the least OD value. Fermencin SA715 production increased with increase in pH reaching a maximum value of 14.02 mm at pH 6 and then declined afterwards. OD increased between pH 3 and 5 after which decline in OD occurred (pH 6–9). As the initial pH of the uninoculated broth increased so did the final pH of the culture.

**Fig. 9:**
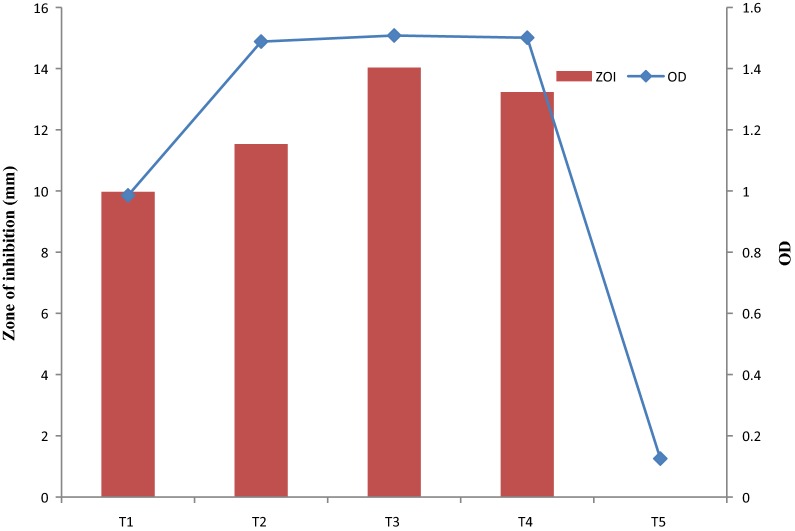
Effect of temperature on bacterial growth and production of fermencin SA715. **T1** (25 °C), **T2** (32 °C), **T3** (37 °C), **T4** (40 °C), **T5** (46 °C)

ZOI obtained at all temperatures were significantly different (p < 0.05) (Fig. 9). The highest ZOI was obtained at 37 °C. Fermencin SA715 production did not occur at 46 °C. OD at 37 °C and 40 °C were not significantly different. OD value obtained at 40 °C was not significantly different from that obtained at 32 °C. OD at 25 °C was significantly higher than 46 °C (the least). There were variations in the final pH of the broth cultures incubated at the different temperatures.

### Scale up of fermencin SA715 production using batch fermentation

**Fig. 10:**
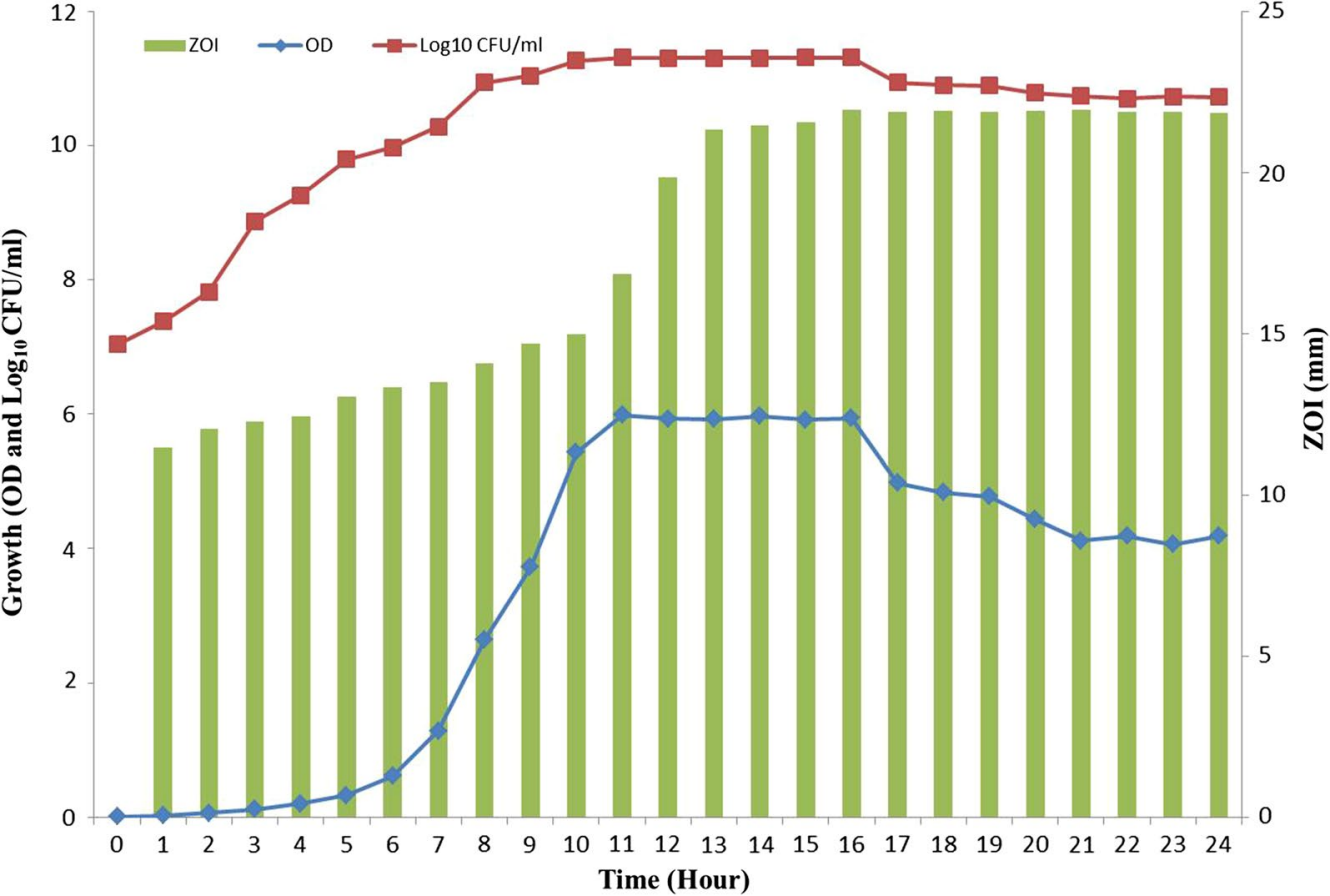
Scale up and enhanced production of fermencin SA715. Bacteriocin was produced in a bioreactor at pH 6.5

Growth of *L. fermentum* GA715 in S11 (SGSL containing 5% sucrose as carbon source) at bioreactor conditions of 37 °C, stirrer speed of 200 rpm and a constant pH value of 6.5 was much higher (maximum OD = 5.984) (Fig. 10) than that observed under shake flask culture conditions (maximum OD = 1.636) (Fig. 4). The same observation was made for fermencin SA715 production (Fig. 10). Growth of the bacteriocin producer was in four phases namely lag phase, log phase, stationary phase and death phase. *L. fermentum* GA715 has a specific growth rate of 0.72/h and a doubling time of 0.96 h. Bacteriocin production started after 1 h of incubation and peaked after 16 h corresponding to late stationary phase of growth of the producer (Fig. 10).

### Biopreservation of banana

**Fig. 11:**
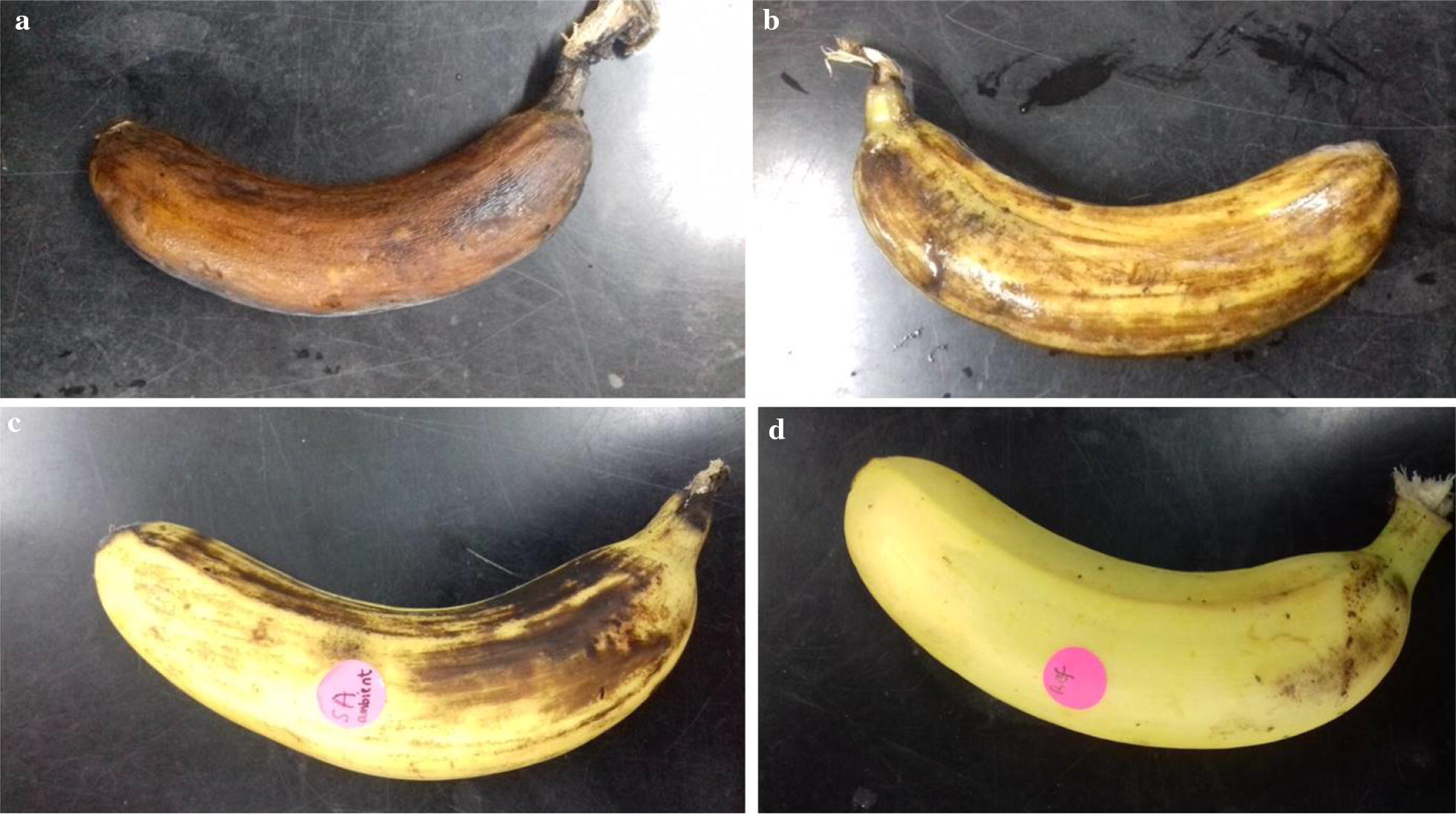
Appearance of banana after 5 days of storage at different conditions. **a** Nonbacteriocin-treated stored at ambient condition, **b** nonbacteriocin-treated stored at refrigeration condition, **c** fermencin SA715-treated stored at ambient condition, **d** fermencin SA715-treated stored at refrigeration condition

Total bacterial count and LAB count of 4.00 × 10^7^ CFU/ml and 2.10 × 10^3^ CFU/ml (0.005% of total bacterial count), 3.70 × 10^5^ CFU/ml and 9.4 × 10^2^ CFU/ml (0.254% of total bacterial count), 2.36 × 10^3^ CFU/ml and 1.20 × 10^3^ CFU/ml (50.85% of total bacterial count), 2.14 × 10^3^ CFU/ml and 1.31 × 10^3^ CFU/ml (61.21% of total bacterial count) were obtained for nonbacteriocin-treated sample stored at ambient condition, non-bacteriocin-treated sample stored at refrigeration condition, fermencin SA715-treated sample stored at ambient condition and fermencin SA715-treated sample stored at refrigeration condition respectively (Table 5). The shelf life of nonbacteriocin-treated sample stored at ambient condition, non-bacteriocin-treated sample stored at refrigeration condition, fermencin SA715-treated sample stored at ambient condition and fermencin SA715-treated sample stored at refrigeration condition are 3, 5, 6 and 9 days respectively. An inverse relationship occurred between total bacterial count and shelf life of banana with a Pearson correlation coefficient value of r = − 0.737 whereas a direct relationship was observed between percentage of LAB and shelf life (r = 0.866). Percentage of non-LAB (NLAB) and shelf life had a Pearson correlation coefficient value of − 0.866 indicating an inverse relationship. Changes in organoleptic properties occurred earlier in nonbacteriocin-treated banana (Fig. 11).

**Table 5:** Effect of fermencin SA715 on surface bacterial count and shelf life of banana

Bacteriocin	Bacterial count (CFU/ml) after 5 days of storage				Shelf-life (days)	
	A		R		A	R
	Total	LAB	Total	LAB		
F	2.36 × 10^3^	1.20 × 10^3^	2.14 × 10^3^	1.31 × 10^3^	6	9
C	4.00 × 10^7^	2.10 × 10^3^	3.70 × 10^5^	9.4 × 10^2^	3	5

Fermencin SA715 was topically applied on fresh bananas and subsequently stored at different conditions. Total bacterial count, LAB count, morphological changes and shelf life were measured

F, fermencin SA715; C, negative control; A, ambient condition; R, refrigeration condition

## Discussion

The presence of *L. fermentum* GA715 in goat milk indicates that it is a component of the goat milk microflora. The fact that it is bacteriocinogenic suggests it plays a significant role to eradicate spoilage bacteria thereby preserving the milk quality. Its biopreservative potential is made more evident due to the inhibitory activity of its bacteriocin (fermencin SA715) towards some food spoilage bacteria namely *Listeria monocytogenes*, *Bacillus cereus*, *Staphylococcus aureus*, *Micrococcus luteus*, *Escherichia coli* and *Pseudomonas aeruginosa*. *L. fermentum* GA715 may find future application in in situ preservation of milk and milk products. *L. fermentum* has been isolated from various sources including human vagina [43], human colon [44], human milk [45], dairy products [46], fermented foods [47] and cheeses [48]. Based on available literature, no bacteriocin-producing *L. fermentum* has been isolated from goat milk. Bacteriocinogenic strains of *L. fermentum* are quite rare. We report for the first time, a bacteriocin-producing strain of *L. fermentum* isolated from goat milk.

A combination of HIC, solid-phase extraction and RP-HPLC was sufficient for purification of the bacteriocin to homogeneity. The bacteriocin-producing potential of *L. fermentum* remains hugely untapped. Studies on purification and characterization of bacteriocins from *L. fermentum* are quite scarce. Establishing a purification approach would help in the future industrial production of this bacteriocin. We have successfully purified and accurately determined (using a combination of HIC, solid-phase extraction and RP-HPLC) the molecular weight of bacteriocin produced by *L. fermentum* GA715. This bacteriocin has a unique molecular weight compared to all reported bacteriocins isolated from *L. fermentum.* Hence, it was named fermencin SA715.

Fermencin SA715 is a broad-spectrum bacteriocin with high inhibitory potency against food-borne pathogens and food spoilage bacteria. Broad spectrum of antibacterial activity is one of the important criteria for selection of bacteriocin for use in the biopreservation of foods [22, 49]. The broad antibacterial spectrum of fermencin SA715 well positions it as a good candidate for preservation of variety of foods. It is strongly inhibitory towards *Staphylococcus aureus*, *Pseudomonas aeruginosa* and *Escherichia coli*. This has been observed for some *L. fermentum*-produced bacteriocins [37, 38]. However, in addition to these pathogens, it was also highly potent against *Listeria monocytogenes*, *Bacillus cereus*, *Corynebacterium* spp. *Streptococcus sanguinis* and *Streptococcus equisimilis*. This is a unique characteristic found in fermencin SA715. Other than fermencin SA715 only one other *L. fermentum*-derived bacteriocin (fermentcin B) has been reported to possess inhibitory activity towards *Micrococcus luteus* [40] however, the potency of fermencin SA715 against *Micrococcus luteus* is much higher than that of fermentcin B. The fact that the antibacterial spectrum of fermencin SA715 includes both Gram-positive and Gram-negative bacteria adds credence to its biopreservative potential.

Fermencin SA715 possesses such a high thermal stability that even heating at 100 °C did not produce any significant drop in its activity. Although residual antibacterial activity was higher in the acidic pH range than at alkaline pH values, overall it exhibited a wide range of pH stability. Considering the harsh conditions of food processing, high thermal and wide range of pH stability are major criteria in the selection of candidate bacteriocins for biopreservation of processed foods [50, 51]. The proteinaceous nature of fermencin SA715 was revealed by the significant drop in activity after treatment with proteinase K, peptidase, trypsin, α-chymotrypsin and protease. Sensitivity of the bacteriocin to proteases is a desirable characteristic since it reduces its chances of inhibiting beneficial components of the gut microbiota thereby enhancing the safety of the bacteriocin [51, 52]. Moreover, degradation of bacteriocin by proteases reduces the time of interaction between fragments of a given bacteriocin and its target thereby decreasing the possibility of resistance development [53]. Its application in the treatment of gut infection would require approaches such as bioengineering and encapsulation into nanoparticles to make it resistant to proteases of the gut [4, 54, 55].

Two key modes of action have been reported for LAB bacteriocins. These include pore formation and inhibition of cell wall synthesis [12, 56, 57]. Pore formation assay using SYTOX^®^ Green dye hinges on the principle that formation of pores would lead to increase in fluorescence due to leakage of intracellular DNA whereas lack of increased fluorescence is indicative of the absence of pore formation. Fermencin SA715 did not induce pore formation in the selected target as no increased fluorescence was observed. Pore formation occurs via interaction of bacteriocin with lipid II (a cell wall precursor). This is the most common mechanism of action of LAB bacteriocins. However, some LAB bacteriocin can also interact with lipid I or II leading to inhibition of cell wall synthesis without membrane permeabilization [12]. We propose that fermencin SA715 acts against its bacterial targets via this mechanism.

Fermencin SA715 is a cell–wall associated bacteriocin due to the fact that higher antibacterial activity was detected in the cell extract than in the cell-free supernatant. It is thought that this attribute is an adaptive mechanism to enhance niche competition. In the midst of complex microbial communities where nutrients are limiting, bacteriocin accumulation on the cell wall could provide added protection thereby enhancing nutrient uptake, utilization and growth of the producer. This trait has been reported for crispacin A produced by *Lactobacillus crispatus* JCM 2009 [58] and bovicin HC5 produced by *Streptococcus bovis* HC5 [59]. Future studies would involve developing a method for dissociating fermencin SA715 from the surface of the producer as a means of increasing its concentration in the culture supernatant. This would help in its future industrial scale production.

Optimization of production is an important step in the industrial scale up of any product [64] and therefore production optimization of fermencin SA715 was carried out. Media compositions had a significant impact on the production of fermencin SA715. A newly synthesized medium (SGSL) was produced. This medium supported higher biomass accumulation and fermencin SA715 production than in MRS. Two other media (TPYGMA and TPYGCaT80) were therefore tried. However, both did not support bacteriocin production which is attributed to poor cell growth caused by the two media components that did not seem to support the physiological demands of the strain. Apparently they lack essential nutrients required for growth and fermencin SA715 production. Media type has been reported to have effect on growth and bacteriocin production [60, 61]. The growth of *L. fermentum* GA715 was in three stages: lag, log and stationary phases. Slowdown in growth after 10 h could be due to increase in acidity of the medium over time culminating in the attainment of stationary phase. Extending the time taken to attain stationary phase by adding a buffering agent may have a positive impact on biomass accumulation and bacteriocin titer. This information is important with regards to the industrial production of fermencin SA715.

In order to understand the role played by each nutrient in SGSL with regards to biomass accumulation and bacteriocin production, various derivatives lacking only one nutrient were produced. Removal of MnSO_4_ had the most significant impact on growth of the bacteriocin producer. Furthermore there was no bacteriocin production in the absence of MnSO_4_. This clearly shows that MnSO_4_ is a very vital requirement for fermencin SA715 production. Its impact can be attributed to enhancing the activity of enzymatic machineries required for synthesis, maturation of fermencin SA715 and growth of the producer. The next most significant nutrient is yeast extract. It is thought that yeast extract contain essential amino acids that drive the growth and production of fermencin SA715. Of the three nitrogen sources (tryptone, peptone and yeast) yeast extract had the most significant impact on growth and production of bacteriocin while peptone had the least impact. Addition of yeast extract enhanced bacteriocin production in a strain of *Lactococcus lactis* [62]. Tween 80, MgSO_4_, NaCl, ascorbic acid and sodium citrate are required for enhanced growth and bacteriocin production. Tween 80 caused fourfold increase in bacteriocin production by *Lactococcus lactis* subsp. *cremoris* J46 [63]. Tween 80 enhanced the activity of bovicin HC5 [59]. Optimum bacteriocin production by *Lactobacillus* sp. MSU3IR was recorded in the presence of Tween 80 [64]. The effect of Tween 80 could be associated with disaggregation and desadsorption of fermencin SA715. Sodium chloride and MgSO_4_ are required for the synthesis of bacteriocin by *Lactococcus lactis* [62]. Bacteriocin ST28MS production by *Lactobacillus plantarum* was facilitated in the presence of ascorbic acid [65].

Growth and production of fermencin SA715 was best in the presence of sucrose as a carbon source. This shows the preference of the bacteriocin producer cells for sucrose. Maltose is the next preferred carbon source followed by glucose, fructose, lactose and finally galactose. Its preference for sucrose suggests that it possesses a transport system and metabolic pathway for its efficient uptake and utilization. In addition, the resulting fructose after hydrolysis bypasses the isomerization step required in the glycolytic breakdown of glucose thus making the process faster. Sucrose is the best carbon source for a bacteriocinogenic strain of *Lactococcus lactis* [62].

Growth at 0.5% and 1% sodium chloride concentrations were better than at 0.02% but fermencin SA715 production was unaffected. Impact on growth can be attributed to enhanced metabolic activities. At higher sodium chloride concentration (2% and 4%), growth was impaired due to stress induced by the salt (Fig. 7). Reduced bacteriocin production at these concentrations (2% and 4%) could be due to interference with bacteriocin induction suggesting that fermenting SA715 production is inducible, mediated by a quorum-sensing mechanism. Increase in sodium chloride concentration from 0.02 to 0.5% and 1% did not significantly alter fermencin SA715 production. Increasing sodium chloride concentration could have positive or deleterious effects on growth and bacteriocin production depending on the LAB strain. High sodium chloride concentration (2%) did not affect growth but reduced curvacin A production in a strain of *Lactobacillus curvatus* [66]. Sodium chloride influenced the production of bacteriocin by *Lactobacillus plantarum* ST202Ch in a concentration-dependent manner [67]. Enhanced growth of LAB at sodium chloride concentrations in the range of 1–2% has been reported [68–71].

pH of media had a significant impact on bacteriocin production. There was no fermencin SA715 production at pH value of 3 because of impaired growth. Although optimal growth of *L. fermentum* GA715 occurred at pH 4–5 and the unadjusted MRS, the optimum pH for fermencin SA715 production was pH 6–7. Optimum growth does not always imply optimum bacteriocin production. The effect of pH on fermencin SA715 production could be attributed to a plethora of factors. These include its influence on the growth of *L. fermentum* GA715, activity and solubility of the bacteriocin and adsorption of the bacteriocin to the cell wall of the producer. Bacteriocin production by *Lactobacillus casei* was optimal at pH 7 [71].

Lack of fermencin SA715 production at an incubation temperature of 46 °C was mainly due to growth suppression. Although growth at 37 °C and 40 °C were not significantly different, optimum incubation temperature for the production of fermencin SA715 was 37 °C. This difference may be due to the influence of temperature on the induction of bacteriocin production and solubility of the bacteriocin.

Having established that fermencin SA715 was optimum in S11 (SGSL containing 5% sucrose as carbon source), at a temperature and pH values of 37 °C and 6–7 respectively, it was imperative to scale up bacteriocin production in a bioreactor as this is an important industrialization step. Batch fermentation at a constant pH value of 6.5 resulted in much enhanced growth of the producer and fermencin SA715 production. Without maintaining pH auto acidification of the growth medium due to lactic acid production occurred resulting in lowering of pH, consequently suppressing the growth of *L. fermentum* GA715 and fermencin SA715 production. Enhanced production of nisin was achieved at a constant pH of 6.8 [72]. Buildup of lactic acid in growth medium can impair growth of LAB. This is due to the fact that lactic acid can diffuse across bacterial cytoplasmic membrane and dissociate resulting in acidification of the cytoplasm and eventual death if the pH drop is more than the cytoplasmic buffering capacity [72–75]. This problem can be circumvented by addition of alkali to neutralize acidity due to ionization of lactic acid [74, 75]. It is pertinent to add that in addition to the fact that bacteriocin production is better in S11 (SGSL containing 5% sucrose as carbon source) than in MRS it is also a cheaper growth medium. These findings would help in the future industrial production of fermencin SA715.

Banana is one of the most consumed and economically important fruit product in the tropical and subtropical regions of the world [76, 77]. It contains antioxidants, carbohydrates, calcium and potassium [78]. As a perishable and climacteric crop it has a short shelf life [26]. Preserving fresh banana is quite challenging [79]. Various chemical and physical approaches are employed in the preservation of bananas [78, 80, 81]. However, consumer requisition for food containing biopreservatives and less chemical preservatives stimulated the search for natural products that can be used for biopreservation [1]. In a recent study combined application of phenylurea and gibberellins was effective at extending the shelf life of banana [76]. Although the bioprotective capabilities of several bacteriocins have been reported [82–84], no report has been published for fresh bananas. Topical application of fermencin SA715 extended the shelf life of fresh banana. Shelf life extension was due to decrease in total bacterial count, decrease in percentage of NLAB and increase in percentage of LAB (Table 5). It can be deduced that fermencin SA715 decreased the population of pathogenic and spoilage bacteria on the surface of the banana. Moreover, it had a positive effect on the population dynamics of the surface microflora such that decrease in spoilage bacteria enhanced the growth of beneficial LAB strains leading to shelf life extension. This conforms to a report that the shelf life and microbial safety of minimally processed apples increased due to the application of *Lactobacillus plantarum* [85]. Furthermore, bacteriocin treatment followed by refrigeration improved the microbiological quality and tripled the shelf life of the banana. These findings pave the way for future ex situ application of fermencin SA715 in the biopreservation of bananas.

## Conclusions

*Lactobacillus fermentum* GA715 produces a novel bacteriocin called fermencin SA715. Enhanced production occurred in a newly synthesized growth medium namely S11. Optimum condition for the production of fermencin SA715 has been established with the view of enhancing future commercialization. A scale up approach has been established which would help in future industrial production of fermencin SA715. A novel approach for the biopreservation of fresh banana using fermencin SA715 has been demonstrated. Moreover, fermencin SA715 displayed unique and interesting characteristics which includes broad spectrum of antibacterial activity, high potency and excellent physicochemical attributes that can be exploited in its future application as a food biopreservative.

## Abbreviations

ACN: acetonitrile
CFS: cell-free supernatant
HIC: hydrophobic interaction chromatography
LAB: lactic acid bacteria
NLAB: non-lactic acid bacteria
OD: optical density
PBS: phosphate buffered saline
RP-HPLC: reversed-phase HPLC
ZOI: zone of inhibition
